# Innovo GenMax MTB-RIF/INH: a moderate-complexity automated NAAT for rapid simultaneous detection of *Mycobacterium tuberculosis* complex and rifampin/isoniazid resistance

**DOI:** 10.3389/fcimb.2025.1600170

**Published:** 2025-06-30

**Authors:** Xichao Ou, Bing Zhao, Huiwen Zheng, Ruida Xing, Qian Sun, Zhonghua Qin, Lixia Zhang, Kai Cui, Yuanyuan Song, Yang Zheng, Yang Zhou, Shengfen Wang, Hui Xia, Yanlin Zhao

**Affiliations:** ^1^ National Key Laboratory of Intelligent Tracking and Forecasting for Infectious Diseases, National Center for Tuberculosis Control and Prevention, Chinese Center for Disease Control and Prevention, Beijing, China; ^2^ Beijing Key Laboratory of Pediatric Respiratory Infection Diseases, Key Laboratory of Major Diseases in Children, Ministry of Education, National Clinical Research Center for Respiratory Diseases, Laboratory of Respiratory Diseases, Beijing Pediatric Research Institute, Beijing Children’s Hospital, Capital Medical University, National Center for Children’s Health, Beijing, China; ^3^ Microbial Inspection Department, Changping District Center for Disease Control and Prevention, Beijing, China; ^4^ Center for Accurate Detection of Tuberculosis, Tianjin Haihe Hospital, Tianjin, China

**Keywords:** tuberculosis, rifampin, isoniazid, drug resistance, rapid molecular diagnosis

## Abstract

**Objective:**

Given the increase of treatment failure, relapse and acquired resistance observed in isoniazid (INH) resistance, there is an urgent to improve rifampin (RIF) -priority based diagnostic strategies. Therefore, we evaluated the performance of Innovo GenMax MTB-RIF/INH (GenMax), a moderate- complexity automated nucleic acid amplification test (NAAT), for detecting Mycobacterium tuberculosis complex (MTBC) and resistance to RIF and INH.

**Methods:**

Analytical sensitivity (limit of detection, LOD) was determined using serial dilutions of *Mycobacterium tuberculosis* H37Rv (ATCC 27249) strains. Diagnostic accuracy was assessed in clinical sputum specimens against microbiological reference standards (MRS: positive by smear microscopy, culture or Xpert MTB/RIF for diagnosis of TB) and phenotypic drug susceptibility testing (DST). Discordant results were resolved by sequencing resistance genes (*IS6110*, *rpoB*, *katG*, *inhA*, *ahpC*) and follow-up diagnosis results.

**Results:**

GenMax demonstrated a calculated LOD of 8.8 CFU/mL (95% CI: 7.4-11.4) for MTBC, 674.1 CFU/mL (95% CI: 578.8-923.5) for RIF resistance, and 747.3 CFU/mL (95% CI: 613.7-1081.3) for INH resistance. In clinical evaluation, the sensitivity and specificity for MTBC detection were 97.52% (95% CI: 92.38–99.36) and 93.65% (95% CI: 88.91–96.53), respectively. For RIF and INH resistance, sensitivities were 88.46% (95% CI: 68.72–96.97) and 85.19% (95% CI: 65.39–95.14), with specificity of 92.42% (95% CI: 82.50-97.18) and 94.12% (95% CI: 84.86-98.10).

**Conclusion:**

Innovo GenMax MTB-RIF/INH is a rapid and automated assay with high sensitivity for MTBC detection, suitable for decentralized settings. While its performance for RIF/INH resistance detection is competitive with existing assays, its sensitivity remains gaps relative to WHO targets. Further optimization, particularly through expanded probe coverage, is needed to bridge this gap and ensure reliable detection in clinical settings.

## 1 Introduction

Tuberculosis (TB), caused by *Mycobacterium tuberculosis* complex (MTBC), remains a significant public health challenge in China, which is among the 30 high-burden TB countries globally. In 2023, China was estimated with approximately 741,000 incident TB cases, including 29,000 cases of multidrug-resistant or rifampicin-resistant TB (MDR/RR-TB) (World Health Organization, 2024b). However, only 52.9% MDR/RR-TB cases were identified by genotypic or phenotypic methods in 2023 (World Health Organization, 2024b). Moreover, isoniazid (INH) resistance testing is often restricted to rifampicin-resistant cases in China, leaving isoniazid mono-resistant TB under-diagnosed and mismanaged (Liu et al., 2024). Previous reports revealed that isoniazid resistance affects approximately 11% TB patients nationally (Zhao et al., 2012), while 7.8% culture positive cases were isoniazid-resistant/rifampin-susceptible (H^r^-R^s^) (Liu et al., 2024). As isoniazid resistance is associated with an increase of treatment failure, relapse and acquired resistance (Gegia et al., 2017; Menzies et al., 2009), it is crucial to improve rifampin-priority based diagnostic strategies, requiring decentralized, rapid diagnostic tools capable of detecting both rifampin and isoniazid resistance, which is critical for clinical decisions making and improved treatment outcomes in Hr-Rs TB patients.

The InnowaveDX MTB/RIF (InnowaveDX company, Suzhou, China), a real-time PCR assay, targeting MTB and rifampicin resistance-determining region (RRDR), demonstrated high accuracy for MTBC and rifampin resistance detection (Deng et al., 2023; Fan et al., 2023). Innovo GenMax MTB-RIF/INH (GenMax), an updated version, expands diagnostic capabilities by incorporating isoniazid resistance with mutations in the promoter of *inhA* (-18–5), the promoter of *ahpC* (-15–2), and the 315 codon of *katG.* This study aimed to evaluate the performance of Innovo GenMax MTB-RIF/INH, which are designed to be implemented in peripheral laboratories with limited infrastructure.

## 2 Methods

### 2.1 Participants

Sputum samples were collected from 310 patients presenting with TB symptoms or abnormal chest x-ray results at Tuberculosis Prevention and Control Institute of Changping District and Tianjin Haihe Hospital from February to July in 2024, which were stored in biobank in National Tuberculosis Reference Laboratory. Patients who have received ≥ 2 weeks of anti-tuberculosis treatment at enrollment were excluded to avoid selective pressure induced new resistance mutations, ensure reliable phenotype-genotype correlation, avoid false positives from non-viable bacterial nucleic acids, and patients lacked sputum production capacity were also excluded.

### 2.2 Microbiological reference standard

All sputum samples were subjected to Ziehl-Neelsen staining directly to confirm acid-fast bacilli rapidly (Steingart et al., 2006). Then the specimens were digested in N-acetyl-L-cysteine NaOH-Na citrate (1.5% final concentration) and neutralized with phosphate buffer (PBS, 0.067 mol/L, pH = 7.4), followed by incubation into the Bactec MGIT 960 system for 6 weeks mycobacterial culture to improve diagnostic accuracy (Hanna et al., 1999). Positive cultures were further subjected to MPT64 antigen detection (Park et al., 2009). For Xpert MTB/RIF assay, a rapid nucleic acid amplification test recommended by the WHO for initial diagnosis of TB with drug resistance, 1 mL of the processed specimen was mixed with 2 mL sample reagent, incubated at room temperature for 10 min, and then transferred into cartridges for analysis using the GeneXpert instrument (Boehme et al., 2010). Microbiological reference standard (MRS) was defined as positive by smear microscopy, culture or Xpert MTB/RIF for the diagnosis of TB (World Health Organization, 2024a).

### 2.3 Phenotypic drug susceptibility testing

The 1% proportion method on solid L-J medium was used for testing of susceptibility to rifampin and isoniazid. Critical concentration was 40 μg/mL for rifampin and 0.2 μg/mL for isoniazid as recommended by WHO (WHO, 2008).

### 2.4 Innovo GenMax MTB-RIF/INH

**Figure 1:**
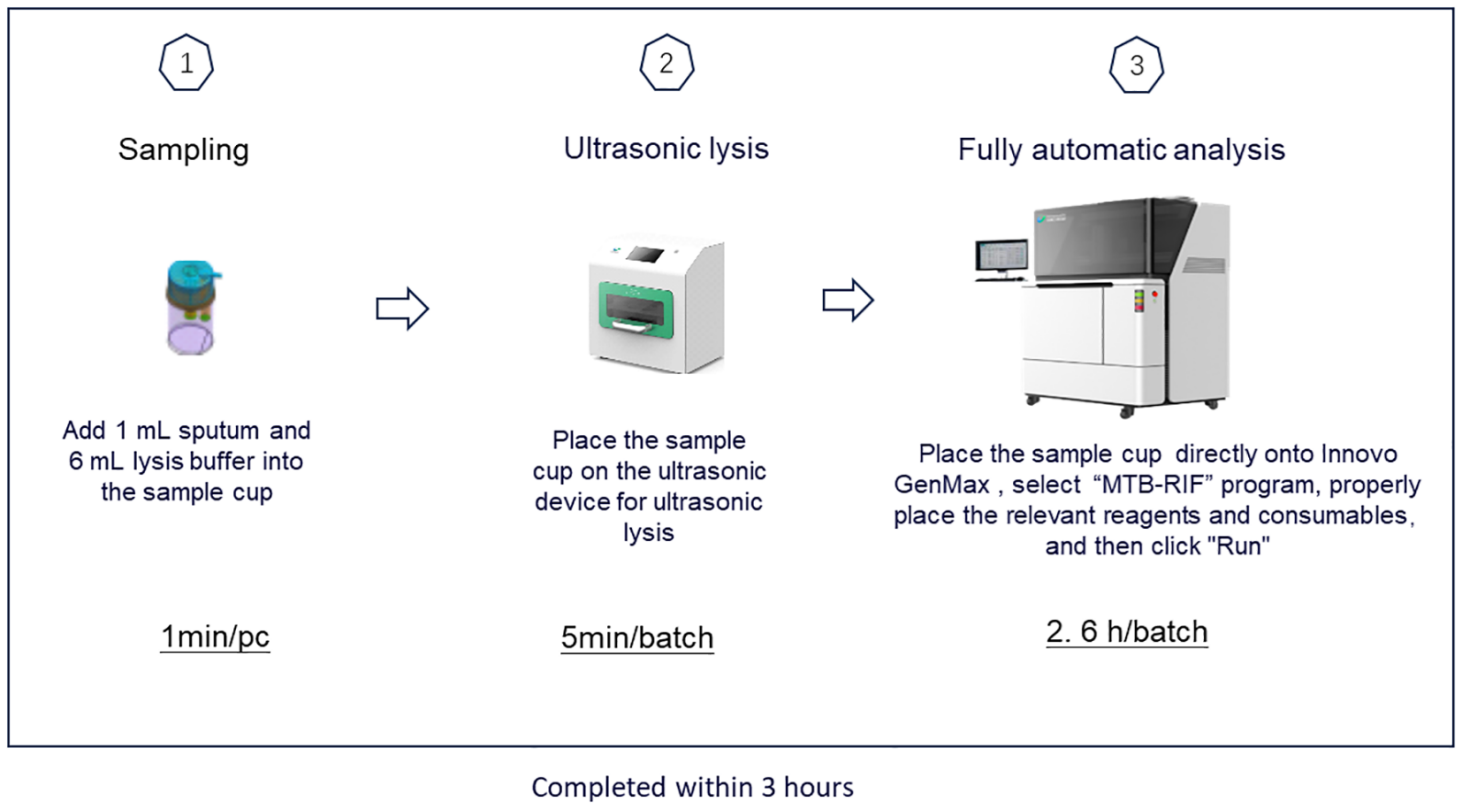
The operation process of GenMax.

GenMax was operated according to the manufacture’s instruction. Briefly, 1mL sputum was added into the pretreatment tube with 6 mL lysis solution. The pretreated mixed sputum was proceeding with ultrasonic instrument for 5 minutes for DNA extraction, and then uploaded into the GenMax instrument installed with specific software. The results were read and interpreted according to the manual. The whole procedure takes around 3 hours (**Figure 1**).

### 2.5 Sequencing of fragments of IS6110, rpoB, ahpC, inhA, and katG gene

Boiling method was used for crude DNA extraction from sputum for sequencing. The DNA was amplified with primers shown in **Table 1** and subjected to sequencing for *IS6110, rpoB, ahpC, inhA, and katG* gene fragments. The sequencing results were compared with the H37Rv sequence.

**Table 1:** Primers used for sequencing of *IS6110, rpoB, ahpC, inhA*, and *katG* gene.

Primers	Sequences(5’-3’)
IS6110-Seq-F	CACGACCGAAGAATCCGCTG
IS6110-Seq-R	GCGGCTGATGTGCTCCTTGA
rpoB-Seq-F	CCGGTGGAAACCGACGACAT
rpoB-Seq-R	CACGTCGCGGACCTCCAGC
ahpC-Seq-F	CACCGAGACCGGCTTCCGA
ahpC-Seq-R	ACCCGCCACTTGCCTGGGT
inhA-Seq-F	CTGAGTCACACCGACAAACG
inhA-Seq-R	TCACATTCGACGCCAAACAG
katG-Seq-F	GGTCACACTTTCGGTAAGA
katG-Seq-R	GCCGTCCTTGGCGGTGTA

### 2.6 Limitation of detection and analytic specificity

The limit of detection (LOD) was determined by diluted *Mycobacterium tuberculosis* H37Rv (ATCC 27249) strain with 1×10^3^ CFU/mL at a series of concentrations (0.125 CFU/mL, 1.25 CFU/mL, 2.5 CFU/mL, 5 CFU/mL, 10 CFU/mL, 20 CFU/mL). The LOD for rifampicin and isoniazid resistance was performed by diluted with mono-rifampin resistant or mono-isoniazid resistant MTB strain with 1 × 10^5^ CFU/mL at a series of concentrations (2000 CFU/mL, 1000 CFU/mL, 500 CFU/mL, 250 CFU/mL, 25 CFU/mL). Each sample with a defined dilution was tested 20 replicates. In addition, the analytical specificity was tested using (approximately 10^4^ CFU/ml) strains of 17 different species of nontuberculous mycobacteria (NTM) and 12 other common bacteria or virus (**Table 2**).

**Table 2:** Strains used in assessment of analytical specificity.

No.	Bacteria/virus	No.	Bacteria/virus	No.	Bacteria/virus
1	M.kansasii	11	M. chelonae	21	S. epidermidis
2	M. marinum	12	M.foruitum	22	Cryptococcus
3	M. terrae	13	M.smegmatis	23	Influenza A virus
4	M. triviale	14	M.abscessus	24	Influenza B virus
5	M.ulcerans	15	M. gastri	25	S. aureus
6	M.gordonae	16	M. intracellulare	26	Nocardia
7	M.xenopi	17	M. phlei	27	P. aeruginosa
8	M.avium	18	S.pneumoniae	28	Candida albicans
9	M.scrofulaceum	19	Haemophilus influenzae	29	Human Parainfluenza Viruses1/2/3
10	M. szulgai	20	Escherichia coli		

### 2.7 Data analysis

SPSS version 20.0 (IBM, Chicago, IL) software was used. The diagnostic accuracy of the GenMax assay was described as point estimates and 95% confidence intervals (95% CIs). The consistency between GenMax and MRS for MTB detection, and accordance between GenMax and phenotypic DST for rifampin and isoniazid resistance was conducted with Kappa analysis. For calculation of the LOD values, the percentages of the replicates resulting in successful TB detection and rifampin/isoniazid resistance were calculated at each input CFU concentration in suspensions. Probit analysis was used to generate the curve through the tested concentrations, and lower and upper 95% confidence intervals (95% CIs).

## 3 Results

### 3.1 The limit of detection and analytic specificity

**Figure 2:**
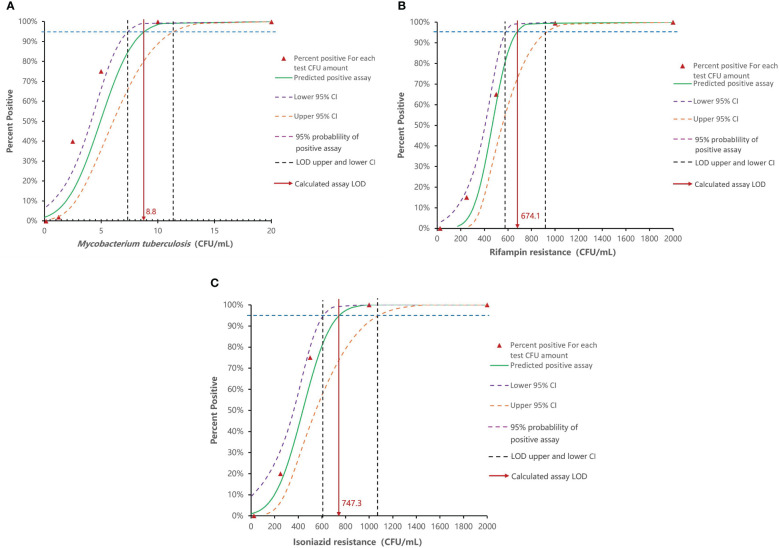
Analytical sensitivity of Innovo GenMax MTB-RIF/INH. **(A)** MTB detection limit (8.8 CFU/mL, 95% CI: 7.4–11.4). **(B)** Rifampin resistance detection (674.4 CFU/mL, 95% CI: 578.8- 923.5). **(C)** Isoniazid resistance detection (747.3 CFU/mL, 95% CI: 613.7-1081.3).

GenMax demonstrated 100% accuracy in detecting the target MTB strain across all tested samples, with a detection limit as low as 10 CFU/mL. At dilutions below 10 CFU/mL, correct detection rate decreased to 45% for 5 CFU/mL, 10% for 2.5 CFU/mL, and 5% for 1.25 CFU/mL (**Table 3**). The calculated limitation of detection (LOD) was 8.8 CFU/mL (95% CI 7.4-11.4). Correct detection of rifampin mono-resistant strains by GenMax was 100% down to 1×10^3^ CFU/mL, which decreased to 90% for 5×10^2^ CFU/mL, and 30% for 2.5×10^2^ CFU/mL. The calculated rifampin resistance detection limit was 674.1 CFU/mL (95% CI: 578.8- 923.5). As was the case with isoniazid resistance detection limit, 100% corrective detection was down to 1×103 CFU/mL and 95% for 5×102 CFU/mL. The calculated detection limit of isoniazid resistance was 747.3 CFU/mL (95% CI: 613.7-1081.3) (**Figure 2**). For specificity, none of the tested NTM, bacteria or virus strains were detected as *Mycobacterium tuberculosis.*

**Table 3:** The limit of detection of MTB, rifampin-resistance, and isoniazid-resistance.

MTB		Rifampin mono-resistance		Isoniazid mono-resistance	
Conc. (CFU/mL)	Detection rate n/N (%)	Conc. (CFU/mL)	Detection rate n/N (%)	Conc. (CFU/mL)	Detection rate n/N (%)
20	20/20 (100)	2×103	20/20 (100)	2×103	20/20 (100)
10	20/20 (100)	1×103	20/20 (100)	1×103	20/20 (100)
5	9/20 (45)	5×102	18/20 (90)	5×102	19/20 (95)
2.5	2/20 (10)	2.5×102	6/20 (30)	2.5×102	9/20 (45)
1.25	1/20 (5)	2.5×101	0/20 (0)	2.5×101	0/20 (0)
0.125	0/20 (0)	—		—	

### 3.2 Diagnostic accuracy of GenMax for pulmonary TB compared with MRS

A total of 310 cases with presumed pulmonary TB were analyzed. 121 cases were microbiological confirmed by smear microscopy, culture or Xpert MTB/RIF. The sensitivity and specificity of GenMax for the detection of MTBC were 97.52% (95% CI: 92.38-99.36) and 93.65% (95% CI: 88.91-96.53) relative to the MRS, respectively. The PPV and NPV of GenMax was 90.77% (95% CI: 84.10-94.93) and 98.33% (95% CI: 94.82-99.57), respectively (**Table 4**). Among 12 MRS-negative/GenMax-positive samples, *IS6110* sequencing successfully confirmed GenMax results in 8 cases (66.7%), while 4 samples failed of sequencing due to insufficient DNA concentration in the specimen. Follow-up data revealed that 6/12 were bacteriologically confirmed cases, 4/12 cases had previous TB history, and 2 cases lacked subsequent clinical results. 3 MRS-positive/GenMax-negative samples were all XpertMTB/RIF positive.

**Table 4:** Diagnostic accuracy of GenMax for detection of pulmonary TB.

Method		MRS		Sensitivity (%, 95% CI)	Specificity (%, 95% CI)	PPV (%, 95% CI)	NPV (%, 95% CI)	Accordance (%, 95% CI)	Kappa value
		Positive	Negative						
GenMax	Positive	118	12	97.52 (92.38-99.36)	93.65 (88.91-96.53)	90.77 (84.10-94.93)	98.33 (94.82-99.57)	95.16 (92.17-97.05)	0.900
	Negative	3	177						

MRS, microbiological reference standard; PPV, positive predicative value; NPV, negative predicative value.

### 3.3 Diagnostic accuracy of GenMax for pulmonary TB compared with XpertMTB/RIF

When conducting a head-to-head comparison between GenMax and XpertMTB/RIF, the accordance rate was 93.87% (291/310), with kappa value 0.872 (**Table 5**). Among 16 specimens showing Xpert MTB/RIF-negative/GenMax-positive discordant results, MTBC were confirmed in 4 cases through culture. Subsequent *IS6110* sequencing were performed on 12 smear-negative and culture-negative samples, yielding positive results in 8 samples, while sequencing failed in 4 specimens due to insufficient DNA concentration in sputum. Follow-up results showed that 6 of these 12 initially microscopy/culture-negative cases were bacteriologically confirmed during subsequent following-up. 4/12 cases had a previous TB history, while follow-up information was unavailable for 2 cases.

**Table 5:** Head-to-head comparison between GenMax and Xpert MTB/RIF for detection of MTBC.

Method		Xpert MTB/RIF		Positive consistency rate (%, 95% CI)	Negative consistency rate (%, 95% CI)	Accordance rate (%, 95% CI)	Kappa value	*P* value
		Positive	Negative					
GenMax	Positive	114	16	97.44 (92.13-99.34)	91.71 (86.65-95.03)	93.87 (90.63-96.04)	0.872	<0.001
	Negative	3	177					

### 3.4 Diagnostic accuracy of GenMax for detection of rifampin and isoniazid resistance

92 phenotypic susceptibility results were available for comparison of the diagnostic accuracy of rifampin resistance (**Table 6**). The sensitivity of GenMax and Xpert MTB/RIF in detection of rifampin resistance was 88.46% (95% CI: 68.72-96.97) and 84.62% (95% CI: 64.27-94.95), respectively, with the same specificity of 92.42% (95% CI: 82.50-97.18). 3 samples with GenMax-susceptible/phenotypic-resistant were consistently classified as susceptible by both Xpert MTB/RIF and *rpoB* sequencing. Conversely, among five specimens showing GenMax-resistant/phenotypic-susceptible, sequencing revealed RIF resistance-associated with mutations in all cases. A total of 95 cases underwent parallel phenotypic DST and GenMax test. When compared against phenotypic DST, GenMax demonstrated 85.19% (95% CI: 65.39-95.14) sensitivity (23/27) and 94.12% (95% CI: 84.86-98.10) specificity (64/68) for INH resistance detection. The overall concordance rate between GenMax and phenotypic DST reached 91.58% (95% CI: 83.76-95.52). Notably, genetic sequencing of the 8 discordant cases revealed complete alignment with GenMax results (8/8).

**Table 6:** Diagnostic accuracy of GenMax and Xpert MTB/RIF for detection of Rifampin and isoniazid resistance.

Method		Phenotypic DST		Sensitivity (%, 95%CI)	Specificity (%, 95%CI)	Accordance (%, 95%CI)	Kappa value	P value
		Resistant	Susceptible					
Rifampin resistance detection								
GenMax	Resistant	23	5	88.46 (68.72-96.97)	92.42 (82.50-97.18)	91.30 (83.76-95.52)	0.790	<0.001
	Susceptible	3	61					
Xpert MTB/RIF	Resistant	22	5	84.62 (64.27-94.95)	92.42 (82.50-97.18)	90.22 (82.45-94.77)	0.762	<0.001
	Susceptible	4	61					
Isoniazid resistance detection								
GenMax	Resistant	23	4	85.19 (65.39-95.14)	94.12 (84.86-98.10)	91.58 (84.26-95.67)	0.793	<0.001
	Susceptible	4	64					

## 4 Discussion

To facilitate accurately and rapidly diagnosis of pulmonary tuberculosis, novel diagnostics with improved sensitivity are urgently needed (Seki et al., 2018; MacLean et al., 2020). The GenMax provides an option in automated nuclei acid amplification tests (NAATs) for TB diagnosis and resistance detection, particularly in decentralized settings. This study demonstrated high sensitivity for detecting MTBC and its capacity to simultaneously identify resistance to rifampin and isoniazid, two cornerstone drugs in TB treatment.

GenMax exhibited a limit of detection (LOD) of 8.8 CFU/mL for MTBC, surpassing InnowaveDX MTB/RIF (LOD: 9.6 CFU/mL) (Deng et al., 2023), Xpert MTB/RIF (LOD: 131 CFU/mL) (Helb et al., 2010) and Ultra (LOD: 16 CFU/mL) (Chakravorty et al., 2017), though it should be noted that this value was derived solely from testing diluted bacterial suspensions, which has not been validated in sputum specimens spiked with known bacterial loads. The assay’s sensitivity drops to 45% at 5 CFU/mL emphasized optimizing sample processing steps (e.g., pre-enrichment or centrifugation) to concentrate low-abundance targets could improve sensitivity. Moreover, a high diagnostic sensitivity (97.52%) to some extent also reflects a lower LOD of GenMax, although such sensitivity may be also influenced by factors such as the bacterial load in included cases, the preparation of the specimens, and the amplification efficiency of the assay, etc (Dinnes et al., 2007; Chakravorty et al., 2017). The sensitivity for MTBC detection approached the optimal sensitivity requirements (≥95%) on sputum-based assays outlined in the WHO’s Target Product Profile (TPP) (World Health Organization, 2024c), positioning it as a robust tool for paucibacillary samples, such as those from pediatric or HIV/TB co-infected patients. Future studies should expand clinical validation to include larger cohorts of TB cases to confirm robustness. The diagnostic sensitivity is superior to Xpert MTB/RIF Ultra, which demonstrates ~90% sensitivity (World Health Organization, 2024c). GenMax’s automated workflow and shorter turnaround time (~3 hours) make it particularly advantageous in resource-limited settings where skilled personnel and infrastructure are scarce.

GenMax demonstrated sensitivities of 88.46% for RIF resistance and 85.19% for INH resistance. While these values fall short of WHO TPP targets (≥95% for RIF, ≥90% for INH) (World Health Organization, 2024c), they remain competitive with first-generation molecular RIF/INH assays. For instance, in China the GenoType MTBDR assay achieves ~91% sensitivity for RIF resistance but only ~80% for INH resistance (Sun et al., 2019). Similarly, the Genechip shows comparable performance with 88% sensitivity for RIF resistance and 80% for INH resistance detection (Pang et al., 2013). Notably, RIF resistance is predominantly mediated by *rpoB* mutations, whereas INH resistance involves complex mechanisms. The lower sensitivity for INH resistance likely stems from its genetic complexity, involving mutations in *katG*, *ahpC-inhA* promoter and other unknown mechanisms (Liu et al., 2022; WHO, 2023). GenMax’s targeted approach covers some particular regions, such as RRDR for RIF resistance*, katG* codon 315, promoter region of *inhA* and *ahpC* for INH resistance detection, but may miss uncommon variants, necessitating expanded probe coverage or supplemental sequencing. Importantly, sequencing confirmed GenMax results in all discordant cases (8/8), underscoring its reliability in detecting known resistance-associated mutations. However, phenotypic DST discrepancies (e.g., 3 GenMax-susceptible/phenotypic-resistant RIF cases) suggest that rare *rpoB* mutations outside the rifampicin resistance-determining region (RRDR) or heteroresistance may contribute to false negatives of this GenMax assay. Therefore, in high-risk clinical scenarios (e.g., treatment failure cases or contacts of known DR-TB patients), GenMax should be used in conjunction with phenotypic DST or WHO-recommended molecular tests to minimize the risk of missed resistance detection.

The WHO TPP emphasizes the need for rapid, user-friendly, and cost-effective diagnostics in decentralized settings (WHO, 2024). GenMax meets these criteria through its almost automated system (hands-on time <10 minutes) and minimal infrastructure requirements. Results are typically available within 3 hours. This important feature can potentially result in dramatically reduced turnaround times for MTB and resistance to rifampin and isoniazid. Its ability to concurrently detect MTBC, RIF, and INH resistance in a single test streamlines workflows, reducing delays in initiating appropriate therapy. This is critical in China, where only half of MDR/RR-TB cases are currently detected (WHO, 2024), and INH mono-resistance remains underdiagnosed due to limited testing access and RIF resistance prioritized diagnostic algorithm.

This study has several limitations. Firstly, the sample size for drug resistance testing was relatively small (92 cases for rifampin and 95 cases for isoniazid), necessitating validation through multicenter studies and geographically diverse cohorts to confirm its applicability across varying *Mycobacterium tuberculosis* strains and resistance patterns. Secondly, the research focused solely on pulmonary tuberculosis cases; future investigations should evaluate the efficacy of GenMax in extrapulmonary and pediatric tuberculosis samples, where bacterial loads are typically lower. Finally, a comprehensive cost-effectiveness analysis is essential to justify its scalability in resource-limited healthcare systems.

## 5 Conclusion

The Innovo GenMax MTB-RIF/INH assay is a promising tool for rapid, decentralized TB diagnosis and resistance screening, which is critical for clinical decisions making and improved treatment outcomes. While its MTBC detection performance aligns with WHO targets, sensitivity gaps for RIF/INH resistance underscore the need for iterative optimization. Strategic integration with existing technologies and expanded clinical validation will maximize its impact on TB control, particularly in high-burden settings like China.

## Data Availability

The original contributions presented in the study are included in the article/supplementary material. Further inquiries can be directed to the corresponding authors.
